# Bacterial antibiotic resistance development and mutagenesis following exposure to subinhibitory concentrations of fluoroquinolones *in vitro*: a systematic review of the literature

**DOI:** 10.1093/jacamr/dlaa068

**Published:** 2020-09-30

**Authors:** Carly Ching, Ebiowei S F Orubu, Indorica Sutradhar, Veronika J Wirtz, Helen W Boucher, Muhammad H Zaman

**Affiliations:** 1 Department of Biomedical Engineering, Boston University, Boston, MA, USA; 2 Institute for Health System Innovation & Policy, Boston University, Boston, MA, USA; 3 Department of Global Health, Boston University School of Public Health, Boston, MA, USA; 4 Division of Geographic Medicine and Infectious Diseases, Tufts Medical Center, Boston, MA, USA; 5 Howard Hughes Medical Institute, Boston University, Boston, MA, USA

## Abstract

**Background:**

Understanding social and scientific drivers of antibiotic resistance is critical to help preserve antibiotic efficacy. These drivers include exposure to subinhibitory antibiotic concentrations in the environment and clinic.

**Objectives:**

To summarize and quantify the relationship between subinhibitory fluoroquinolone exposure and antibiotic resistance and mutagenesis to better understand resistance patterns and mechanisms.

**Methods:**

Following PRISMA guidelines, PubMed, Web of Science and Embase were searched for primary *in vitro* experimental studies on subinhibitory fluoroquinolone exposure and bacterial antibiotic resistance and mutagenesis, from earliest available dates through to 2018 without language limitation. A specifically developed non-weighted tool was used to assess risk of bias.

**Results:**

Evidence from 62 eligible studies showed that subinhibitory fluoroquinolone exposure results in increased resistance to the selecting fluoroquinolone. Most increases in MIC were low (median minimum of 3.7-fold and median maximum of 32-fold) and may not be considered clinically relevant. Mechanistically, resistance is partly explained by target mutations but also changes in drug efflux. Collaterally, resistance to other fluoroquinolones and unrelated antibiotic classes also develops. The mean ± SD quality score for all studies was 2.6 ± 1.8 with a range of 0 (highest score) to 7 (lowest score).

**Conclusions:**

Low and moderate levels of resistance and efflux changes can create an opportunity for higher-level resistance or MDR. Future studies, to elucidate the genetic regulation of specific resistance mechanisms, and increased policies, including surveillance of low-level resistance changes or genomic surveillance of efflux pump genes and regulators, could serve as a predictor of MDR development.

## Introduction

Antibiotic resistance (AR) is a growing global health threat. Resistant bacterial infections can set back and undermine treatments that we rely on, including cancer chemotherapy, joint replacement surgery and organ transplantation. To improve interventions and policies that aim to reduce AR development, it is critical to understand the drivers of resistance and the underlying scientific mechanisms of resistance evolution. Bacteria are often exposed to sublethal or subinhibitory concentrations of antibiotics. This occurs in the environment due to agricultural activities and wastewater treatment and run-off^1^^,^^2^ and in the clinic due to incorrect therapy, poor adherence and poor-quality medicines.^3–6^ Subinhibitory concentrations are not lethal for bacteria but can still incur stress and put selective pressure on bacteria.^6^ There are currently a few narrative literature reviews on subinhibitory antibiotic exposure and their impact on bacterial resistance.^7–10^ Andersson *et al*.^6^ provide theoretical background and focus broadly on three major aspects (selection dynamics, genotypic and phenotypic variability and signalling molecules), highlighting key studies for all classes of antibiotics.

Among the situations in which subinhibitory antibiotic exposure occurs, medicine quality is often understudied and overlooked. This is important to note because medicine quality is a growing problem. In low- and middle-income countries (LMICs), which are greatly impacted by AR, the observed failure rate for antibiotics to meet quality standards is reported to be about 7% or higher.^11^ Substandard medicines are authorized products that fail to meet quality standards or specifications, primarily through low active pharmaceutical ingredient (API) content or APIs that are not released effectively in the body and are predicted to impact AR.^11–13^ In addition to substandard drugs, falsified drugs are those with deliberately misrepresented contents.^11^

Our systematic review was performed using preferred reporting items for systematic reviews and meta-analyses (PRISMA) guidelines. Narrative literature reviews have an increased risk of bias by presenting only a selection of the literature without systematically appraising the quality of the publications.^14^ A systematic review provides a comprehensive overview of the evidence and importantly includes risk-of-bias assessment. However, it has very limited use for basic science and *in vitro* studies.^14–16^

This aim of this review was to comprehensively summarize and quantify the impact of subinhibitory fluoroquinolone exposure on bacterial AR development and mutagenesis *in vitro*, in order to reveal trends in resistance patterns and mechanisms. Fluoroquinolones are a widely used class of antibiotics in both humans and animals.^17^^,^^18^ Furthermore, we sought to identify gaps in knowledge and assess the current dialogue about medicine quality among studies, which we believe is an underappreciated factor in AR development. This can help design and ensure appropriate interventions to prevent resistance development.

## Methods

The PRISMA guidelines were applied to review experimental microbiological data to investigate and quantify the impact of subinhibitory fluoroquinolone exposure on AR selection and development and mutagenesis.

The full protocol for the systematic review has been published.^19^ We summarize the main search strategy, selection criteria and data extraction and analysis below.

### Search strategy and selection criteria

PubMed, Web of Science and Embase were searched for primary experimental studies from earliest available dates through to 2018 for studies in which bacteria were exposed to subinhibitory concentrations of fluoroquinolones with a defined search strategy based on keywords and MeSH terms.^19^ This search was performed on 16 January 2019. To define the search and outcomes being extracted, we applied a population intervention comparator outcome study (PICOS) search tool for inclusion and exclusion criteria.^19–21^ To be eligible for inclusion, studies had to contain primary experimental evidence that investigated resistance or mutant frequency/mutation rate of any bacteria after exposure to subinhibitory concentrations of second- to fourth-generation fluoroquinolones. No language limitation was placed and papers not in English were translated.

Titles and abstracts of all retrieved citations were screened for duplicates and relevance using Rayyan QCRI.^22^ Full articles that passed abstract screening were checked for eligibility. This was performed by two independent researchers (C.C. and E.S.F.O.). Bibliographies of identified papers and papers that had cited key studies were searched for additional records. Records were managed through reference management software Endnote and Mendeley.

### Data extraction

Each eligible paper was evaluated and outcomes and study variables were extracted to a standardized table. For the main outcome of AR, the minimum and maximum reported change in MIC of the fluoroquinolone that the bacteria were exposed to, relative to the parental strain, was extracted for each test within a study. Tests are defined as a given bacterial species–drug combination within a study and, as such, each study may have multiple tests. Each test is aggregated for isolates of the same species and includes both laboratory strains and clinical isolates. Data for change in mutant frequency and mutation rate were also extracted along with data for MDR development. We also determined whether there was discussion of medicine quality or substandard antibiotics. We assessed the reproducibility of the data extraction process through checking concordance between results from the main data extractor (C.C.) with results from a second reviewer (I.S.) for 10% of included studies. The percentage agreement for data extraction between the two raters was 90%. Papers that did not meet the minimum criteria of ability to extract data on methods and results, such as lack of appropriate quantitative numerical data on study outcome, were excluded.

### Data analysis

The details of data analysis, as well as risk of bias, have been previously published.^19^ Pre-specified quantitative subgroup analysis by independent variable (bacterial species, concentration of exposures, antibiotic etc.) and summarization was performed and is presented below.^19^ Evaluation of whether meta-analysis was appropriate occurred after data extraction and synthesis, based on criteria already defined.^19^

Risk of bias was determined using a specifically formulated non-weighted tool,^19^ which incorporated aspects of the SYstematic Review Centre for Laboratory animal Experimentation (SYRCLE)’s risk-of-bias tool for animal studies^23^ and the Effective Public Health Practice Project (EPHPP) quality assessment tool.^24^ Studies were assessed for a series of criteria^19^ in five domains, expanded upon in Table S1 (available as Supplementary data at *JAC-AMR* Online). A risk-of-bias point was assigned for each unmet review criterion within a domain. The more points that were assigned, the higher the risk of bias associated with the study. Thus, a quality or risk-of-bias score of 0 reflects the lowest risk of bias. The overall quality of the body of evidence (meta-bias) was determined using pre-specified criteria,^19^ largely based on grading of recommendations, assessment, development and evaluations (GRADE) guidelines on publication bias.^25^

## Results

**Figure 1.**
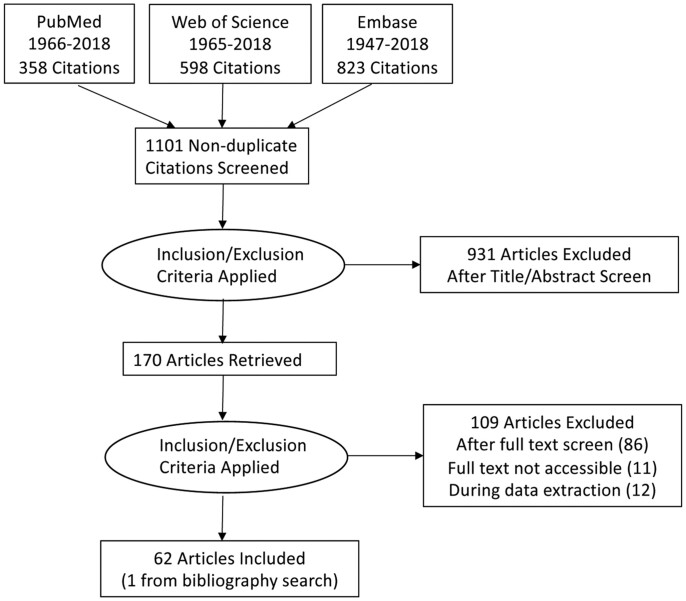
PRISMA flow diagram of the search and review process.

Our search of three databases retrieved 1779 records (Figure 1). After removal of duplicate records, 1101 titles and abstracts remained. After abstract screening by two independent reviewers, 170 records met inclusion criteria. After review of the full texts, 97 records were excluded, with another 12 records excluded during data extraction (Figure 1). A total of 62 papers, including 1 identified from searching bibliographies, were included for data extraction. Out of all the papers, three were not in English (one Spanish, one German and one Russian). We did not perform meta-analyses, mainly because of differences in populations, interventions and methods among studies.

### Outcomes of studies

Of the 62 included studies, 46 (74.2%) reported an outcome on resistance change,^26–71^ 13 (21%) reported on change in mutation rate or mutant frequency^72–84^ and 3 (4.8%) on both.^85–87^ Summarized data are provided in Table S2 and full data extraction results are available upon request.

### Characteristics of studies

Of the 62 included studies, 33 different bacterial species were studied, 11 of which were Gram positive and 22 of which were Gram negative. Of all the included studies, the most commonly studied bacteria were *Pseudomonas aeruginosa* (16/62, 25.8%),^26^^,^^27^^,^^29^^,^^35^^,^^41^^,^^45^^,^^53^^,^^54^^,^^60^^,^^61^^,^^66^^,^^77^^,^^82^^,^^84^^,^^86^^,^^87^*Streptococcus pneumoniae* (12/62, 19.4%)^28^^,^^30^^,^^36^^,^^38^^,^^48^^,^^51^^,^^52^^,^^57^^,^^58^^,^^67^^,^^69^^,^^79^ and *Staphylococcus aureus* (11/62, 17.7%)^27^^,^^37^^,^^42^^,^^44^^,^^47^^,^^67^^,^^72^^,^^75^^,^^77^^,^^80^^,^^81^ (Figure S1A). Multiple studies (11/62, 17.7%)^27^^,^^29^^,^^40^^,^^42^^,^^52^^,^^56^^,^^60^^,^^62^^,^^65^^,^^67^^,^^77^ investigated more than one species of bacteria and most (42/62, 67.7%)^28–30^^,^^33–40^^,^^43^^,^^44^^,^^46–48^^,^^50–52^^,^^54^^,^^57–67^^,^^69^^,^^70^^,^^72^^,^^75^^,^^77^^,^^79–81^^,^^83–85^ tested multiple strains of a specific species. Twenty different fluoroquinolones were studied, with the most prevalent being ciprofloxacin (45/62, 72.6%), followed by levofloxacin (18/62, 29%)^30^^,^^31^^,^^36^^,^^38^^,^^42^^,^^43^^,^^46^^,^^48^^,^^51^^,^^52^^,^^59^^,^^64^^,^^65^^,^^69^^,^^70^^,^^72^^,^^75^^,^^76^ and moxifloxacin (16/62, 25.8%)^34^^,^^38^^,^^43^^,^^51^^,^^52^^,^^56^^,^^58^^,^^59^^,^^62^^,^^65^^,^^67^^,^^69^^,^^70^^,^^72^^,^^75^^,^^76^ (Figure S1B). Multiple studies (29/62, 46.8%)^27^^,^^28^^,^^30^^,^^32^^,^^34^^,^^38^^,^^43^^,^^46^^,^^47^^,^^50–52^^,^^54^^,^^56–60^^,^^63–65^^,^^67–70^^,^^72^^,^^75^^,^^76^^,^^84^ investigated more than one type of fluoroquinolone. The distribution of exposure concentration was 0.1–0.75× the MIC, with 0.5× being the most studied concentration (38/62, 61.3%).^26–28^^,^^30–32^^,^^35^^,^^36^^,^^38–44^^,^^46–50^^,^^52^^,^^55^^,^^57^^,^^58^^,^^60^^,^^63^^,^^66–70^^,^^72^^,^^74^^,^^75^^,^^77^^,^^78^^,^^85^^,^^87^ Among the included studies, the methods involved both antibiotic exposure in liquid (88.5%) or solid medium (11.5%). MIC was determined primarily using CLSI guidelines, including broth microdilution, macrodilution and agar dilution. Some studies also used commercially available strips (Etest, bioMérieux) to determine MIC values. Almost half the studies (28/62, 45.2%) performed passaging that re-adjusted the antibiotic exposure based on the new MIC of the cells.^27^^,^^28^^,^^30^^,^^35–40^^,^^42^^,^^43^^,^^46–48^^,^^50–52^^,^^55–58^^,^^60^^,^^63^^,^^66–70^ Publication dates ranged from 1984 to 2018 (Figure S1C).

### Magnitude of resistance changes

**Figure 2.**
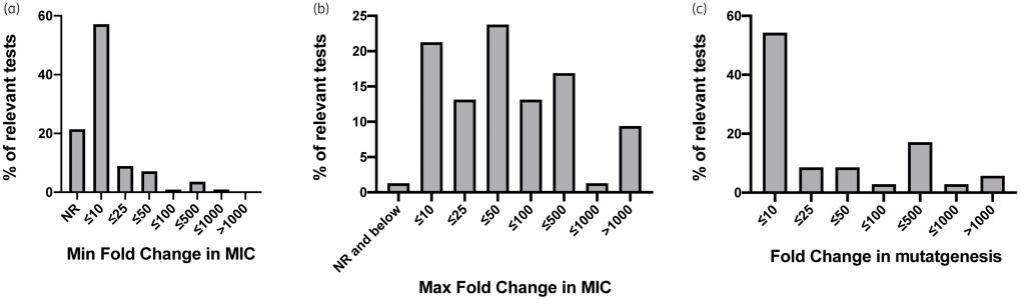
Treatment with subinhibitory concentrations of fluoroquinolones results in increased resistance, largely <50-fold. Distribution of (a) minimum and (b) maximum fold increase in MIC of exposure drug, relative to parental strain, reported among experimental tests (bacteria–drug combination) as a percentage of all relevant tests. For (a) *n *=* *112 and for (b) *n *=* *160. The bounds for each bar are greater than the previous bin’s maximum and less than or equal to the value noted below each bar. (c) Distribution among experimental tests of reported increase in mutagenesis as a percentage of all relevant tests (*n *=* *35). The bounds for each bar are greater than the previous bin’s maximum and less than or equal to the value noted below each bar. NR, no change in resistance.

The fold change in resistance (MIC) against the exposure fluoroquinolone antibiotic ranged from a 2-fold decrease to a >8000-fold increase (Figures 2, S2 and S3). After stratification of the fold change in MIC, we found that for all relevant bacteria–drug tests, the median maximum increase in resistance was 32-fold, with 59.4% of tests reporting maximum resistance increases below 50-fold (*n *=* *160, Figure 2b). The median minimum increase in resistance was 3.7-fold, with 78.6% of relevant tests reporting minimum resistance increases 10-fold and below (*n *=* *112, Figure 2a) and 21.4% of tests reporting no change in resistance. Altogether, these data suggest that upon subinhibitory fluoroquinolone exposure, resistance changes occur but are often low.

**Figure 3.**
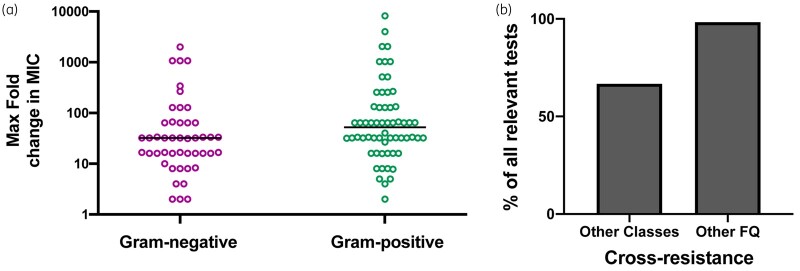
Gram-positive bacteria have higher increases in resistance and subinhibitory fluoroquinolone exposure can lead to cross-resistance to different classes of antibiotics. (a) Spread of maximum fold change in MIC of exposure drug for tests with fluoroquinolone exposure at 0.5× MIC. Each point represents one experimental test (bacteria–drug combination) and is coloured by Gram-positive or Gram-negative. The horizontal line represents the median. (b) Distribution of cross-resistance reported to only fluoroquinolones (FQ) or other classes of antibiotics as a percentage of relevant tests (42 tests for other classes, 120 tests for other FQs). Note that not all tests were tested for the same antibiotics.

There were no notable species-specific trends for change in resistance in relationship to exposure concentration (Figure S2). We did note that the four highest reported changes in resistance at 0.5× MIC exposure belonged to *S. aureus* (Figure S2), from two independent studies.^47^^,^^67^ These two studies passaged clinical isolates at 0.5× MIC (re-adjusted) at 24 h intervals and the bacteria had their QRDR sequenced. Three-quarters of these mutants were triple mutants (increases in MIC of 2048-fold and 8192-fold) while one had a single mutation in *grlA* (4000-fold increase). Gram-positive bacteria overall displayed higher fold changes in resistance compared with Gram-negative bacteria at 0.5× MIC exposure concentration (median maximum increase of 52-fold versus 32-fold, respectively, Figure 3a). We did not see a specific trend by antibiotic (Figure S3).

Almost all tests for MDR^27^^,^^28^^,^^30–32^^,^^34^^,^^37^^,^^38^^,^^40^^,^^43^^,^^45^^,^^47^^,^^51–53^^,^^56–58^^,^^60^^,^^61^^,^^63–72^^,^^77^^,^^84^^,^^86^ showed resistance to other fluoroquinolone antibiotics (98.3% of relevant tests, *n *=* *120, Figure 3b), while 66.7% of tests against other classes of antibiotics showed resistance selection or development to antibiotics such as β-lactams and carbapenems (*n *=* *42, Figure 3b). It should be noted that the panel of drugs tested was not comprehensive or uniform between studies. For mutagenesis, the most frequent changes were 10-fold and below (54.3%, *n *=* *35, Figure 2c). No studies mentioned medicine quality in their introduction or discussion.

### Mechanisms of resistance

**Figure 4.**
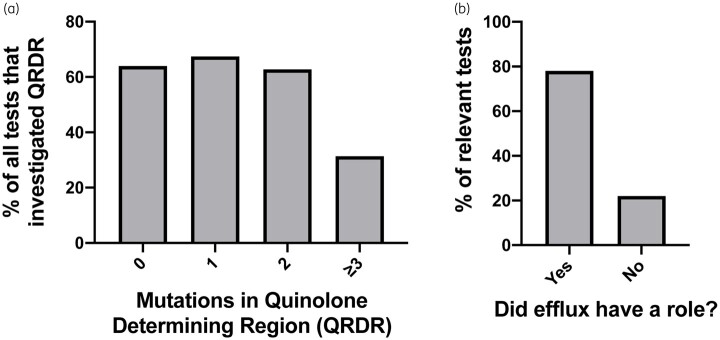
Mechanisms of resistance. (a) Number of mutations in QRDR as a percentage of relevant tests (*n *=* *86) that investigated changes in nucleotide sequence. (b) Percentage of relevant tests that reported a role for efflux in increased AR (*n *=* *50).

Fluoroquinolone resistance typically occurs via mutations in the QRDR composed of DNA gyrase and topoisomerase genes.^15^ A subset of studies tested for mutations in the QRDR of resistant isolates.^26^^,^^28^^,^^30^^,^^32^^,^^34^^,^^36^^,^^38^^,^^41^^,^^43^^,^^45^^,^^47^^,^^49^^,^^51^^,^^52^^,^^56–58^^,^^64^^,^^65^^,^^67^^,^^69^^,^^70^^,^^85^ Of these tests (*n *=* *86), the frequency of zero, one and two mutations among cells exposed to the antibiotic were 64.0%, 67.4% and 62.8%, respectively (Figure 4a). Note that for a test, more than one mutational signature may have been reported. One limitation is that some tests did not look for changes in the full QRDR, but only certain genes. Another mechanism to explain changes in resistance in bacteria that had no QRDR mutations is changes in efflux systems that extrude toxic substances and thus is more broad and less antibiotic-class specific.^88^ Many studies also tested for altered efflux gene expression or activity through experiments with efflux pump inhibitors.^26^^,^^30^^,^^34^^,^^38^^,^^41^^,^^44^^,^^45^^,^^47^^,^^49^^,^^53^^,^^57^^,^^58^^,^^67^^,^^69^^,^^70^^,^^85^ Of the subset of tests for efflux, 78% suggested a role in increased resistance (*n *=* *50, Figure 4b). Most did not identify specific efflux pumps. For those that did, the Mex multidrug efflux system was involved in *P. aeruginosa*,^26^^,^^41^^,^^45^ the NorA efflux system in *S. aureus*^44^ and the PatA/PatB quinolone efflux transporter in *S. pneumoniae*.^38^

### Time dependence

To determine the relationship between exposure time and resistance, we plotted the number of passages as a variable versus fold change in resistance for all studies that tested fluoroquinolone exposure at 0.5× MIC. The mean fold change continued to increase before starting to show a slight decrease after 15 passages (Figure S4). A limitation for these data is that the passages were not uniform and some experiments had a selection cut-off.

### Risk of bias among individual studies

Assessing the quality and risk of bias of each study, we found that the bias score or quality score ranged from 0 (highest quality) to 7 (lowest quality) and the mean ± SD quality score for all studies was 2.6 ± 1.8. The most common criteria that led to a reduction of quality score were lack of clarity about replication of experiments (44/62), incubation conditions (40/62) and control experiments (32/62). To assess reproducibility, a subset of studies was assessed by two independent reviewers. After discussion, discrepancies were resolved. Full risk-of-bias scores are available upon request.

### Meta-bias

We evaluated the outcomes described in included papers by negative result (no change or negative change in outcome) and positive result (positive change in outcome). Only one study contained only a negative result.^53^ Fourteen out of 62 studies reported negative and positive results. Forty-seven of 62 studies reported only positive results. We did not observe any potential lag bias in time of publication (Figure 2c). Of the 62 included studies, three research groups (same corresponding author) had multiple papers (seven papers,^30^^,^^51^^,^^52^^,^^57^^,^^58^^,^^69^^,^^70^ three papers^26^^,^^45^^,^^86^ and two papers,^44^^,^^67^ respectively). For the seven papers from the same group,^30,51,52,57,58,69,70^ five of them reported negative and positive results. For funding sources, 17/62 studies had industry or pharmaceutical funding while 21/62 papers had no funding reported. Of the 17 papers that had industry funding, 8 of them reported negative and positive results. For change in resistance, there were 68 tests for Gram-negative bacteria and 92 tests for Gram-positive bacteria. For mutagenesis, there were 16 tests with Gram-negative bacteria and 19 tests with Gram-positive bacteria.

## Discussion

This review presents a systematic quantitative analysis of 62 papers that investigated subinhibitory exposure to fluoroquinolones and bacterial AR and mutagenesis, from the earliest available dates for PubMed, Embase and Web of Science through to 2018. Our review found that the individual studies included in our review had low bias and that publication bias among all studies was undetected.

Our results show that from the early development of fluoroquinolones, exposure to subinhibitory levels of these drugs has been able to select for bacterial AR to the selecting fluoroquinolone. This resistance is partly explained by changes to target mutations and is also often due to changes in efflux, which is a broader mechanism of resistance (Figure 4). Furthermore, as a collateral consequence, resistance to other fluoroquinolones and unrelated classes of antibiotics also develops (Figure 3b). Our findings show that an increase in resistance occurs for multiple bacteria and multiple antibiotics (Figures S2 and S3).

We did not find any notable bacteria- or antibiotic-specific trends in relationship to magnitude of resistance development. *S. aureus* had the highest fold change in resistance and, consistent with this, Gram-positive bacteria overall had higher fold changes in resistance compared with Gram-negative bacteria (Figures 3a and S2). Fluoroquinolone resistance is more likely to occur in Gram-positive bacteria because many of these bacteria, including *S. aureus*, have lower inherent susceptibility. These bacteria only need one or two mutations to become clinically resistant compared with Gram-negative bacteria, which typically require more.^89–91^ Subinhibitory exposure led to target mutations and, notably, changes in efflux (Figure 4). After exposure, the number of target QRDR mutations ranged from zero to five mutations (Figure 4a). Interestingly, at least three mutations were required to achieve CLSI-classified clinical resistance in *Escherichia coli*.^90^^,^^91^ Furthermore, the bacterial cells with no target mutations represent potentially novel or broader mechanisms of resistance. Numerous studies identified changes in efflux in resistant mutants after exposure to subinhibitory fluoroquinolone concentrations (Figure 4b); this includes mutants that do not have target mutations. It has been observed that high-level clinical resistance does not typically occur from overexpression of MDR efflux pumps alone but is associated with highly resistant clinical isolates.^88^ Furthermore, a study found that clinical isolates of *P. aeruginosa* were enriched in mutations in an efflux regulator that cause low-level, but not clinical, AR and ensured bacterial survival in antibiotic-treated hosts.^92^ Future studies of which specific efflux pumps are involved and how this is regulated will be important to fully understand the evolution of resistance.

Notably, after subinhibitory fluoroquinolone exposure, clinical resistance may not be achieved. For example, the CLSI clinical breakpoint for ciprofloxacin for *E. coli* is 1 mg/L^93^ whereas one study found that the MIC of susceptible clinical isolates ranged from 0.004 to 0.032 mg/L,^94^ which corresponds to 31.25- to 250-fold increases. The majority of maximum fold changes reported were below 50-fold increases, with minimum fold changes below 10-fold increases (Figure 2). Specifically, maximum increases ranged from 10- to 128-fold for *E. coli*. Most clinical testing reports resistance based on the breakpoint value. Therefore, while percentage clinical resistance for different bacterial species can be tracked using hospital antibiograms and retrospective chart reviews, the absolute change in resistance levels (or MIC) may not routinely be collected. Moreover, it is also difficult to determine this change in resistance without information about the parental strain. Smaller resistance increases observed after subinhibitory antibiotic exposure would thus be missed and the evolution of resistant clinical isolates is not fully tracked. This is important as studies suggest that low-level resistance allows bacteria to survive and gain additional mutations under stress, which can include further antibiotic exposure.^95^ This, in turn, leads to higher-level MDR.^96^^,^^97^ Thus, increased surveillance, especially in regions where poor-quality medicines are prevalent, to quantify and track low-level changes in resistance or increased genomic surveillance of efflux pumps and regulators could serve as an important predictor of high-level and MDR development. Indeed, the ESCMID study group for antimicrobial resistance surveillance explicitly called for surveillance of evolving qualitative and quantitative trends of low-level AR as a way to predict high-level resistance.^98^

Our review further aims to bring attention to the underappreciated public health context of medicine quality, as substandard antibiotics often have low API levels or poor dissolution.^11^^,^^12^ Poor-quality (substandard and falsified) medicines could also have increased toxicity and decreased tolerability. After reviewing the evidence, we identified numerous gaps in knowledge. First, no papers included discussion of medicine quality. Thus, the use of poor-quality antibiotics remains an underappreciated social driver of resistance. Moreover, it is critical that public health topics and social drivers of resistance are bridged to experimental studies. Next, most studies reviewed tested the effect of one or two concentrations, primarily at or below 0.5× the MIC. More likely, bacteria in the environment and clinic will be exposed to a broad range of subinhibitory concentrations^6^^,^^95^ and, as such, bacterial responses will vary.

The major limitation of *in vitro* experimental studies is that this does not directly translate to what is observed in the patient, where bioavailability and dosing regimens will differ. Additionally, the immune system also has a role in bacterial survival and antibiotics have been shown to have immunomodulatory effects.^99^^,^^100^ Additionally, *in vitro* experiments use pure APIs, whereas antibiotics used in the environment and clinic will contain other inactive ingredients and have different dissolution kinetics. Future experimental studies include determining bacterial responses to a dynamic range of subinhibitory antibiotic concentrations^95^ and investigating how other aspects of good- and poor-quality drugs (including excipients, impurities or degradation products) impact AR. A complete list of gaps and recommendations is provided in Table 1. Future field studies include comprehensively determining how antibiotic concentrations vary in environmental and clinical conditions. Another gap is that experimental bench research is relatively rarely dissected in systematic reviews in comparison with traditional narrative reviews and, as such, systematic review tools are lacking.^14^ A comprehensive unbiased review of the evidence base is an important tool for basic scientific questions, especially for those with conflicting evidence.

**Table 1.** Gaps in evidence

Gap	Recommendation
Experiments tested limited subinhibitory concentrations.	Perform experiments testing the impact of a wide range of drug concentrations on AMR.
No data on other aspects of substandard antibiotics, such as impurities and degradation products.	Perform experiments testing the impact of impurities and degradation products on AMR.
Little to no mention of medicine quality.	Increase public health context within scientific literature.
Systematic reviews not common for basic science.	Call for more systematic reviews. Develop guidelines, tools and protocols. Incorporate more systematic search strategies for literature reviews.

AR is a complex phenomenon and the impact of medicine quality remains an understudied theme. However, given the complexity, the issue of subinhibitory antibiotic exposure and quality is only part of a much bigger system of drivers that lead to AR^5^ that work in synergy with each other. Therefore, much work remains to both identify and understand different drivers and how they interact with each other. This requires communication and collaboration between multiple different disciplines.

Overall, this review quantifies the evidence base of experimental studies on subinhibitory fluoroquinolone exposure and AR. We find that this type of exposure can lead to selection and development of antibiotic-resistant bacteria. We note that often these changes are low or moderate and thus may be initially missed in the clinic but could still contribute to the evolution of high-level resistance or MDR. We also find that efflux is associated with these resistance changes and is an area for more detailed investigation. We hope our review serves as a stimulus for both systematic basic science reviews of experimental microbiological data and reviews that merge public health and basic science. Altogether, this synthesis of data can serve as support for evidence-based policies regarding surveillance of AR and increased monitoring and surveying of poor-quality antibiotics.

## Supplementary Material

dlaa068_Supplementary_Data
