# Supplementary material for: Bacterial antibiotic resistance development and mutagenesis following exposure to subinhibitory concentrations of fluoroquinolones in vitro: a systematic review of the literature
Source: JAC Antimicrob Resist. 2020 Sep 30;2(3):dlaa068. doi: 10.1093/jacamr/dlaa068 (PMC8210091; doi:10.1093/jacamr/dlaa068)
Supplement: dlaa068_Supplementary_Data [file dlaa068_supplementary_data.docx]

**Supplementary data**

**Table S1. Expanded criteria for Risk of Bias scoring**

|  | Question/Criteria | Scoring | Examples |
| --- | --- | --- | --- |
| Domain 1: Selection and confounding bias | Were the groups compared individually or were differences discussed in the analysis? | If no, 1.  If yes, 0. | No would encompass if, for example, analysis and conclusions about all groups (bacteria, treatment) were not parsed out or individually analyzed. |
|  | Were species and strain details provided? | If no, 1.  If yes, 0. | Genotype is not required, but the source of the strain should be provided. |
|  | Other | If no, 0.  If yes, 1. |  |
| Domain 2:  Study Design/Methods | Are there any discrepancies between methods and in-text? | If no, 0.  If yes, 1. | An example of yes, would be if something is done one way in the methods, but different details of methods are provided in the text or figure captions. Another example would be if what is reported in the results does not match what is in the methods, or there are no matching methods. |
|  | Is the methodological section missing any steps or appropriate detail ? (including but not limited to below) | If no, 0.  If yes, 1. | Steps/Details:  -media used  -temperature  -time  -incubation conditions (static, rolling, shaking, aeration)  - reagents used (antibiotic, buffers, etc. (anything else used for the experiment, etc.)  - concentrations used (for example, if adding ciprofloxacin, how much (either concentration or dilution) was added  - appropriate control experiments (drug-free control)  - replication of experiments (replication per strain)  If NCLSS guidelines are cited and refer to given experiment, the details (media, temperature, time, conditions) are okay. |
| Domain 3: Incomplete outcome data | Is there missing outcome data that was not addressed? | If no, 0.  If yes, 1. | An example of yes, is if the outcome is defined in the methods but not present in the results, or if data is missing from a table or figure and not explained. |
|  | Is the control outcome data mentioned in the paper present? | If no, 1.  If yes, 0. | If the control is mentioned, is this data present or described in the text? |
| Domain 4: Selective outcome reporting | Was all data reported for all conditions or just select/statistically significant results?/Was it clear whether no change results were reported? | If no, 1.  If yes, 0. | For no, based on methods there is a risk that the paper only displays and reports select results with disregard for values that are not significant or results that did not show a change were not quantified, or displayed. |
|  | Was statistical significance noted (if possible)? | If no, 1.  If yes, 0. | If statistical analysis is mentioned, is it noted (is it presented on figures)? When claims are made in the text about strong changes, significance etc., is the data/method for statistical significance present? |
|  | Is the appropriate comparison to baseline provided? | If no, 1.  If yes, 0. | Are the results being compared to the appropriate value (for change in MIC, is the correct baseline MIC being used etc.)? Is the baseline in anyway confounded? |
| Domain 5: Other sources of bias | Was there any Potential bias not covered by other domains. | If no, 0.  If yes, 1. |  |

| **Table S2. Study Details and Outcomes** | | |  |  |  |  |  |  |  |  |  |  |  |  |
| --- | --- | --- | --- | --- | --- | --- | --- | --- | --- | --- | --- | --- | --- | --- |
| Yr/Author (reference) | Bacterial Species | Drugs Tested^0^ | Passag-ing^1^ | Conc.^2^ | Adju-sted^3^ | MIC (mg/L) | Min.  MIC  Change^4^ | Max.  MIC  Change^5^ | No. of QRDR  Mutations^6^ | Effl-ux^7^ | Mutagenesis^8^ | Multidrug Resistance Change^9^ | Drug Quality Discussed | Risk of Bias^10^ |
| ^1^2018/Ahmed | *P. aeruginosa* | CIP | multi | 0.5 | no | 0.094 | NR | 340.4 | 1 | 1 | - | - | No | 1 |
| ^2^2012/Aiassa | *P.mirabilis* | CIP | multi | 0.5 | no | 0.125-2 | 2^a^ | 128^a^ | 0 | 1 | ND  resistance frequency - change not reported (ND) | - | No | 5 |
| ^3^1989/Aldridge | *P. aeruginosa* | LOM | multi | 0.5 | yes | 1 |  | 16 | - | - | - | 1 | No | 3 |
|  | *S. marcescens* | LOM |  | 0.5 |  | 0.5 |  | 8 | - | - | - | 1 |  |  |
|  | *P. mirabilis* | LOM |  | 0.5 |  | 0.5 |  | 2 | - | - | - | 1 |  |  |
|  | *E. cloacae* | LOM |  | 0.5 |  | 0.25 |  | 16 | - | - | - | 1 |  |  |
|  | *K. pneumoniae* | LOM |  | 0.5 |  | 0.25 |  | 2 | - | - | - | 1 |  |  |
|  | *S.aureus* | LOM |  | 0.5 |  | 0.5 |  | 4 | - | - | - | 1 |  |  |
|  | *P. aeruginosa* | NOR |  | 0.5 |  | 0.25 |  | 2 | - | - | - | 1 |  |  |
|  | *S. marcescens* | NOR |  | 0.5 |  | 0.12 |  | 33.3 | - | - | - | 1 |  |  |
|  | *P.mirabilis* | NOR |  | 0.5 |  | 0.12 |  | 16.7 | - | - | - | 1 |  |  |
|  | *E. cloacae* | NOR |  | 0.5 |  | 0.5 |  | 4 | - | - | - | 1 |  |  |
|  | *K. pneumoniae* | NOR |  | 0.5 |  | 0.12 |  | 33.3 | - | - | - | 0 |  |  |
|  | *S.aureus* | NOR |  | 0.5 |  | 1 |  | 8 | - | - | - | 1 |  |  |
| ^4^2007/Avrain | *S. pneumoniae* | CIP | multi | 0.5 | yes | 0.5-1.5 | 2.7 | 5 | 0 | 1 | - | 1 | No | 3 |
|  | *S. pneumoniae* | LEV |  | 0.5 |  | 0.5-0.75 | 3.3 | 5 | 1 | 1 | - | 1 |  |  |
|  | *S. pneumoniae* | MOX |  | 0.5 |  | 0.15 | 3.3 | 26.7 | 1,2 | 0 | - | 1 |  |  |
|  | *S. pneumoniae* | GAR |  | 0.5 |  | 0.05 | 4 | 40 | 0,2 | 0 | - | 1 |  |  |
| ^5^2012/Bai | *E. coli* | ENRO | multi | 0.5 | no | 0.05 |  | 10 | 0 | 1 | - | - | No | 1 |
| ^6^1984/Barry | *﻿K. pneumoniae* | CIP | multi | 0.5 | yes | 0.03 |  | 8.3 | - | - | - | 1 | No | 4 |
|  | *﻿K. pneumoniae* | NOR |  | 0.5 |  | 0.12 |  | 266.6 | - | - | - | 1 |  |  |
|  | *﻿K. pneumoniae* | ENX |  | 0.5 |  | 0.25 |  | 8 | - | - | - | 1 |  |  |
|  | *﻿E. aerogenes* | CIP |  | 0.5 |  | 0.015 |  | 16.7 | - | - | - | 1 |  |  |
|  | *﻿E. aerogenes* | NOR |  | 0.5 |  | 0.12 |  | 16.7 | - | - | - | 1 |  |  |
|  | *﻿E. aerogenes* | ENX |  | 0.5 |  | 0.25 |  | 64 | - | - | - | 1 |  |  |
|  | *﻿E. cloacae* | CIP |  | 0.5 |  | 0.008-0.015 | 16.7 | 31.3 | - | - | - | 1 |  |  |
|  | *﻿E. cloacae* | NOR |  | 0.5 |  | 0.12 |  | 66.6 | - | - | - | 1 |  |  |
|  | *﻿E. cloacae* | ENX |  | 0.5 |  | 0.12-0.25 | 8 | 32 | - | - | - | 1 |  |  |
|  | *﻿P. aeruginosa* | CIP |  | 0.5 |  | 0.03 |  | 33.3 | - | - | - | 1 |  |  |
|  | *﻿P. aeruginosa* | NOR |  | 0.5 |  | 0.5 | 8 | 32 | - | - | - | 1 |  |  |
|  | *﻿P. aeruginosa* | ENX |  | 0.5 |  | 1 |  | 32 | - | - | - | 1 |  |  |
| ^7^2001/Boos | *﻿S. pneumoniae* | CIP | multi | 0.5 | yes | 0.25-1 | 2^a^ | 256^a^ | 0,1,2,3 | ND (can't tell because of starting) | - | 1 | No | 4 |
|  | *﻿S. pneumoniae* | SPAR |  | 0.5 |  | 0.06-0.125 | 32 ^a^ | 128 ^a^ | 0,1,2 | ND (can't tell because of starting) | - | 1 |  |  |
|  | *﻿S. pneumoniae* | GAT |  | 0.5 |  | 0.06-0.25 | 8 ^a^ | 1024 ^a^ | 1,2 | ND (can't tell because of starting) | - | 1 |  |  |
|  | *﻿S. pneumoniae* | MOX |  | 0.5 |  | 0.03-0.125 | 16 ^a^ | 1024 ^a^ | 1,2,3 | ND (can't tell because of starting) | - | 1 |  |  |
|  | *﻿S. pneumoniae* | CLIN |  | 0.5 |  | 0.03-0.125 | 8 ^a^ | 32^a^ | 1,2 | ND (can't tell because of starting) | - | 1 |  |  |
|  | *﻿S. pneumoniae* | GEMI |  | 0.5 |  | 0.015 | 16 ^a^ | 512 ^a^ | 1,2 | ND (can't tell because of starting) | - | 1 |  |  |
|  | *﻿S.pyogenes* | CIP |  | 0.5 |  | 0.125-2 | 4 ^a^ | 128 ^a^ | - | 0 | - | 1 |  |  |
|  | *﻿S.pyogenes* | SPAR |  | 0.5 |  | 0.06-0.5 | 16 ^a^ | 512 ^a^ | - | 0 | - | 1 |  |  |
|  | *﻿S.pyogenes* | GAT |  | 0.5 |  | 0.06-0.5 | 4 ^a^ | 64 ^a^ | - | 0 | - | 1 |  |  |
|  | *﻿S.pyogenes* | MOX |  | 0.5 |  | 0.06-0.25 | 8 ^a^ | 256 ^a^ | - | 0 | - | 1 |  |  |
|  | *﻿S.pyogenes* | CLIN |  | 0.5 |  | 0.03-0.06 | 2 ^a^ | 64 ^a^ | - | 0 | - | 1 |  |  |
|  | *﻿S.pyogenes* | GEMI |  | 0.5 |  | 0.03-0.06 | 8 ^a^ | 64 ^a^ | - | 1 | - | 1 |  |  |
|  | *﻿S.aureus* | CIP |  | 0.5 |  | 0.125-0.25 | 32 ^a^ | 2048 ^a^ | 2,3 | 1 | - | 1 |  |  |
|  | *﻿S.aureus* | SPAR |  | 0.5 |  | 0.06 | 4 ^a^ | 256 ^a^ | 0,2,3 | 1 | - | 1 |  |  |
|  | *﻿S.aureus* | GAT |  | 0.5 |  | 0.06 | 16 ^a^ | 2048 ^a^ | 1,2,3 | 1 | - | 1 |  |  |
|  | *﻿S.aureus* | MOX |  | 0.5 |  | 0.06 | NR ^a^ | 1024 ^a^ | 0,2,3 | -/Nodata | - | 1 |  |  |
|  | *﻿S.aureus* | CLIN |  | 0.5 |  | 0.015-0.03 | NR ^a^ | 128 ^a^ | 0,2 | 1 | - | 1 |  |  |
|  | *﻿S.aureus* | GEMI |  | 0.5 |  | 0.015-0.03 | 8 ^a^ | 8192 ^a^ | 1,2,3 | 1 | - | 1 |  |  |
| ^8^2001/Brook | *B. anthracis* | CIP | multi | 0.5 | yes | 0.5-1 | 8 | 16 | - | - | - | 1,2 | No | 3 |
|  | *B. anthracis* | alatrOFL |  | 0.5 |  |  |  | 7.8 | - | - | - | 1,2 |  |  |
|  | *B. anthracis* | GAT |  | 0.5 |  |  | 32 | 64 | - | - | - | 1 |  |  |
| ^9^2002/Browne | *S. pneumoniae* | LEV | multi | 0.5 | yes | 0.5-2 | NR ^a^ | 16 ^a^ | 0,1,2,3 | 1 | - | 1 | No | 3 |
|  | *S. pneumoniae* | GAT |  | 0.5 |  | 0.125-0.5 | 4 ^a^ | 32^a^ | 1,2,3 | 1 | - | 1 |  |  |
|  | *S. pneumoniae* | MOX |  | 0.5 |  | 0.06-0.125 | NR ^a^ | 33.3^a^ | 0,1,2,3 | 1 | - | 1 |  |  |
| ^10^2003/Browne | *pneumococci* | CIP | multi | 0.25-0.5 | yes | 0.5-32 | 8 ^a^ | 1024 ^a^ | 0,1,2, 3 | 1 | - | 1 | No | 3 |
|  | *pneumococci* | MOX |  | 0.25-0.5 |  | 0.125-4 | 2 ^a^ | 32^a^ | 1,2,3,4 | 1 | - | 1 |  |  |
|  | *pneumococci* | SITA |  | 0.25-0.5 |  | 0.016-0.5 | 4 ^a^ | 266 ^a^ | 0,1,2,3 | 1 | - | 1 |  |  |
|  | *pneumococci* | GAT |  | 0.25-0.5 |  | 0.25-4 | 2 ^a^ | 128 ^a^ | 0,1,2,3 | 1 | - | 1 |  |  |
|  | *pneumococci* | LEV |  | 0.25-0.5 |  | 0.5-16 | 4 ^a^ | 128^a^ | 0,1,2,4 | 1 | - | 1 |  |  |
| ^11^2001/Carman | *B.fragilis group/mixed* | CIP |  | 0.2 | no | 2 |  | 2 | - | - | - | 1 | No | 1 |
| ^12^2015/Chow | *P. aeruginosa* | CIP | multi | 0.1 | no | 0.125 |  | NR | - | - | - | ND | No | 1 |
|  | *P. protegens* | CIP |  | 0.1 |  | 0.25 |  | 10 |  |  | - | ND |  |  |
| ^13^2001/Cottagnoud | *S. pneumoniae* | CIP | multi | 0.5 | yes | 0.5 |  | 16 | 2 | - | - | 1 | No | 4 |
|  | *S. pneumoniae* | TROV |  | 0.5 |  | 0.12 |  | 33.3 | 2 | - | - | 1 |  |  |
| ^14^2010/Dalhoff | *S. aureus* | CIP |  | 0.5 |  | 4-64 | - | - | - | - | 107.9*^a^ | - | No | 3 |
|  | *S. aureus* | LEV |  | 0.5 |  | 4-64 | - | - | - | - | 27.71*^a^ | - |  |  |
|  | *S. aureus* | MOX |  | 0.5 |  | 0.12-32 | - | - | - | - | 3.44*^a^ | - |  |  |
| ^15^1999/Davies | *﻿S. pneumoniae* | CIP | multi | 0.5 | yes | 0.5-2 | NR ^a^ | 16 ^a^ | 0,1,2 | 1 | - | 1 | No | 3 |
|  | *﻿S. pneumoniae* | LEV |  | 0.5 |  | 0.5-1 | 4 ^a^ | 64 ^a^ | 0,1,2 | 1 | - | 1 |  |  |
|  | *﻿S. pneumoniae* | GREP |  | 0.5 |  | 0.06-0.25 | 4 ^a^ | 32 ^a^ | 0,1,2 | 1 | - | 1 |  |  |
|  | *﻿S. pneumoniae* | SPAR |  | 0.5 |  | 0.125-0.25 | 4 ^a^ | 64 ^a^ | 0,1,2 | 1 | - | 1 |  |  |
|  | *﻿S. pneumoniae* | TROV |  | 0.5 |  | 0.06-0.125 | NR ^a^ | 64 ^a^ | 1,2,3 | 1 | - | 1 |  |  |
| ^16^2012/de Souza | *F. nucleatum* | LEV | single | 0.5 | no | 4 |  | 16 ^a^ | - | - | - | 2 | No | 3 |
| ^17^1998/Dessus-  Babus | *C. trachomatis* | OFL | multi | 0.5 | no | 1 |  | 64 | 1 | - | - | 1 | No | 3 |
|  | *C. trachomatis* | SPAR |  | 0.5 |  | 0.03 |  | 1066.7 | 1 | - | - | 1 |  |  |
| ^18^2018/Efimochkina | *C. jejuni* | CIP | multi | ND |  | 0.5 | NR | 2 | ND | - | - | - | No | 7 |
| ^19^2004/Eick | *﻿P. gingivalis* | CIP | multi | 0.25 | no | ﻿0.064–0.25 | ND | ND | ND | 0 | - | 1 | No | 6 |
|  | *﻿P. gingivalis* | GAT |  | 0.25 |  | 0.006-0.023 |  | 333.3 | 0 | 0 | - | 1 |  |  |
|  | *﻿P. gingivalis* | MOX |  | 0.25 |  | 0.006-0.032 | 125 | 5333.3 | 0,1 | 0 | - | 1 |  |  |
| ^20^1991/Ferguson | *P. aeruginosa* | CIP | multi | 0.5 | yes | 0.125-4 | NR | 64 | - | - | - | - | No | 2 |
| ^21^2004/Flatz | *S. pneumoniae* | LEV | multi | 0.5 | yes | 1 |  | 64 | 2,3 | - | - | - | No | 4 |
| ^22^1988/French | *S. aureus* | NOR | multi | ND | yes | <0.125-16 |  | 64***  (mode) | - | - | - | 1 | No | 4 |
| ^23^2018/Frenoy | *E. coli* | NOR |  | ND (defined by MIC) 50ng/mL | no |  | - | - | - | - | 6^b^ | - | No | 1 |
| ^24^1993/Fung-Tomc  Ho  1993/Fung-Tomc | *P. aeruginosa* | CIP |  | 0.5 |  | 0.13 | - | - | - | - | 125**^a^ | 2 | No | 1 |
|  | *P. aeruginosa* | CIP |  | 0.25 |  | 0.13 | - | - | - | - | 185** ^a^ | 2 |  |  |
|  | *P. aeruginosa* | CIP |  | 0.125 |  | 0.13 | - | - | - | - | 7.69** ^a^ | 2 |  |  |
|  | *S. aureus* | CIP |  | 0.125 |  | 0.5-1 | - | - | - | - | 11** ^a^ | 2 |  |  |
|  | *S. aureus* | CIP |  | 0.5 |  | 0.5-1 | - | - | - | - | 255** ^a^ | 2 |  |  |
|  | *S. aureus* | CIP |  | 0.25 |  | 0.5-1 | - | - | - | - | 30**^a^ | 2 |  |  |
|  | *S. aureus* | CIP |  | 0.25 |  | 0.5 | - | - | - | - | 606.1^b^ | - |  |  |
| ^25^2001/Ganiere | *﻿S. intermedius* | ENRO | multi | 0.5 | yes | 0.063-0.25 | 2 | 15.9 | - | - | - | - | No | 2 |
| ^26^2002/ Gautier-Bouchardon | *﻿M. gallisepticum* | ENRO | multi | 0.5 | yes | <0.03-0.06 | 8.3 | 1067 | - | - | - | 0 | No | 2 |
|  | *﻿M.iowae* | ENRO |  | 0.5 |  | <0.03 | 267 | 1067 | - | - | - | 0 | No |  |
|  | *﻿M. synoviae* | ENRO |  | 0.5 |  | 0.5 | NR | 32 | - | - | - | 0 | No |  |
| ^27^2005/Gillespie | *M. fortuitum* | CIP |  | 0.5 |  | 0.12 | - | - | - | - | 91.85**^b^ ^bb^(man to cip) | - | No | 0 |
|  | *M. fortuitum* | CIP |  | 0.25 |  |  | - | - | - | - | 9.53** | - |  |  |
|  | *M. fortuitum* | CIP |  | 0.125 |  |  | - | - | - | - | 4.05** | - |  |  |
| ^28^2010/Gregoire | *﻿P. aeruginosa* | CIP | single | 0.5 | no | 0.25 | 4 | 8 | 0 | 1 | - | - | No | 1 |
| ^29^2006/ Henderson-Begg | *﻿S. pneumoniae* | CIP |  | 0.75 |  | 0.25 | - | - | - | - | 4.07*^a^ ^a^(med.03**ian) | - | No | 1 |
|  |  | CIP |  | 0.5 |  | 0.5 | - | - | - | - | 2.03** |  |  |  |
| ^30^2003/Iwao | *H. pylori* | LEV | multi | 0.5 | yes | 0.39 |  | 4 | - | - | - | - | No | 3 |
|  | *S. aureus* | LEV |  | 0.5 |  | 0.2 |  | 2 | - | - | - | - |  |  |
|  | *E. coli* | LEV |  | 0.5 |  | 0.05 |  | 32 | - | - | - | - |  |  |
| ^31^2003/Jonas | *L. pneumophila* | CIP | multi | 0.5 | yes | 0.015-0.03 | 8 ^a^ | 16 ^a^ | 0,1 | - | - | 1 | No | 5 |
|  | *L. pneumophila* | LEV |  | 0.5 |  | 0.015 | 8 ^a^ | 32 ^a^ | - | - | - | 1 |  |  |
|  | *L. pneumophila* | CLIN |  | 0.5 |  | 0.002-0.004 | 4 ^a^ | 16 ^a^ | - | - | - | 1 |  |  |
|  | *L. pneumophila* | TROV |  | 0.5 |  | 0.004 | 4 ^a^ | 16 ^a^ | - | - | - | 1 |  |  |
|  | *L. pneumophila* | MOX |  | 0.5 |  | 0.015-0.03 | 4 ^a^ | 16 ^a^ | 0,1 | - | - | 1 |  |  |
| ^32^2000/Jones | *S. aereus* | CIP | multi | 0.5 | no | 2-128 | 4 | 8 | 1 | 1 | - | - | No | 1 |
| ^33^2013/ Jørgensen | *P. aeruginosa* | CIP | multi | 0.25 | no | 0.2 | 42.1 ^a^ | 168.4^a^ | 1,2 | 1 | - | 2 | No | 2 |
| ^34^2013/Kaskatepe | *E. coli* | CIP | multi | 0.5 | yes | 0.0078 - 0.25 | NR | 64 | - | - | - | - | No | 1 |
|  | *E. coli* | LEV |  | 0.5 |  | 0.0078-0.5 | NR | 128 | - | - | - | - |  |  |
| ^35^2003/Kim | *S. aureus* | CIP | multi | 0.5 | yes | 0.016-0.063 | 63.5 ^a^ | 4000 ^a^ | 1,2,3 | 1 | - | 1 | No | 3 |
|  | *S. aureus* | GEMI |  |  |  | 0.004-0.031 | 4 ^a^ | 32.4 ^a^ | 0,1,2 | 1 | - | 1 |  |  |
|  | *S. aureus* | SPAR |  |  |  | 0.031-0.25 | 4 ^a^ | 16.1 ^a^ | 1,2 | 1 | - | 1 |  |  |
|  | *S. aureus* | TROV |  |  |  | 0.016-0.063 | 2.0 ^a^ | 31.7 ^a^ | 0,1,2 | 1 | - | 1 |  |  |
| ^36^2004/Koeth | *S. pneumoniae* | LEV | multi | 0.5 | yes | 1-32 | NR | 64 | - | - | - | - | No | 2 |
|  |  |  |  |  |  |  |  |  |  |  |  |  |  |  |
| ^37^1989/Korting | *N. gonorrhoeae* | CIP | multi | 0.5 | yes | 0.0005-0.001 | 32 | 128 | - | - | - | - | No | 2 |
|  |  | ENX |  |  |  | 0.002-0.004 | 250 | 2000 |  |  |  |  |  |  |
| ^38^2006/ Kosowska- Shick | *S. pneumoniae* | MOX | multi | 0.25 | yes | 0.125-4 | NR ^a^ | 32 ^a^ | 0, 1,2,3 | - | - | 1,2 | No | 4 |
|  | *S. pneumoniae* | LEV |  |  |  | 0.5-16 | 2 ^a^ | 32 ^a^ | 2 | - | - | 1 |  |  |
|  | *S. pneumoniae* | CIP |  |  |  | 1--32 | 2 ^a^ | 8 ^a^ | 0,2 | - | - | 1,2 |  |  |
|  | *S. pneumoniae* | GAT |  |  |  | 0.25-8 | NR ^a^ | 16 ^a^ | 0,1 | - | - | 1,2 |  |  |
|  | *S. pneumoniae* | GEMI |  |  |  | 0.016-0.5 | 1.9 ^a^ | 31.3 ^a^ | 0,1,2,3 | - | - | 1,2 |  |  |
| ^39^2008/ Kosowska- Shick | *S. pneumoniae* | LEV | multi | 0.5 | yes | 0.5-16 | 2 ^a^ | 32 ^a^ | 2 | - | - | 1,2 (decrease) | No | 4 |
|  | *S. pneumoniae* | MOX |  | 0.5 |  | 0.125-4 | NR ^a^ | 32 ^a^ | 0,1,2,3 | - | - | 1,2 |  |  |
|  | *H. influenzae* | LEV |  | 0.5 |  | 0.03-8 | NR ^a^ | 33.3 ^a^ | 1,2 | - | - | 1,2 |  |  |
|  | *H. influenzae* | MOX |  | 0.5 |  | 0.03-4 | 2 ^a^ | 16.7 ^a^ | 0,1 | - | - | 1,2 |  |  |
| ^40^2014/Kumari | *P. aeruginosa* | CIP | single | 0.2 | no | 0.25 |  | 0.5 | - | 0 | - | 2 | No | 1 |
| ^41^2009/Mesak | *S. aureus* | CIP |  | ND |  | ND | - |  | - | - | 1.49**^a^ | - | No | 2 |
| ^42^1988/Modak | *P. aeruginosa* | NOR | multi | ND |  | 5 (nmol/mL) |  | 40 | - | - | - | - | No | 7 |
|  | *P. aeruginosa* | PEF |  | ND |  | 5 |  | 40 |  | - | - | - |  |  |
|  | *P. aeruginosa* | OFL |  | ND |  | 5 |  | 100 |  | - | - | - |  |  |
| ^43^2014/Mohamed | *﻿S. pseudintermedius* | CIP | multi | 0.5 | yes | ND | 2 | 8 | - | - | - | - |  | 5 |
| ^44^2002/Morrissey | *C.trachomatis* | CIP | multi | 0.5 | yes | 1  1 | 2 | 128 | 1 | - | - | - | No | 3 |
|  | *C.trachomatis* | OFL |  |  |  | 1 | 2 | 256 | 1 | - | - | - |  |  |
|  | *C. pneumoniae* | MOX |  |  |  | 0.12  2  0.12 |  | 2 | - | - | - | - |  |  |
|  | *C. pneumoniae* | OFL |  |  |  | 2 | 2 | 4 | - | - | - | - |  |  |
|  | *C. pneumoniae* | SPAR |  |  |  | 0.12 | 2 | 133.3 | - | - | - | - |  |  |
| ^45^2000/Nagai | *S. pneumoniae* | CLIN | multi | 0.5 | yes | 0.06-0.5 | 2 ^a^ | 66.7 ^a^ | 0,1,2 | 1 | - | 1 | No | 3 |
|  | *S. pneumoniae* | CIP |  | 0.5 |  | 1-32 | 2 ^a^ | 32 ^a^ | 0,1,3 | 1 | - | 1 |  |  |
|  | *S. pneumoniae* | TROV |  | 0.5 |  | 0.06-2 | 1.92 ^a^ | 133.3 ^a^ | 1,2,3 | 1 | - | 1 |  |  |
| ^46^2001/Nagai | *S. pneumoniae* | GEMI | multi | 0.5 | yes | 0.015-0.25 | 4 ^a^ | 133.3 ^a^ | 0,1,2,3 | 1 | - | 1 | No | 3 |
|  | *S. pneumoniae* | TROV |  | 0.5 |  | 0.06-4 | NR ^a^ | 33.3 ^a^ | 0,1,2,3 | 1 | - | 1 |  |  |
|  | *S. pneumoniae* | CIP |  | 0.5 |  | 0.25-4 | 2 ^a^ | 32 ^a^ | 0,1 | 1 | - | 1 |  |  |
|  | *S. pneumoniae* | GAT |  | 0.5 |  | 0.125-4 | 2 ^a^ | 32 ^a^ | 0,1,2,3,4 | 1 | - | 1 |  |  |
|  | *S. pneumoniae* | MOX |  | 0.5 |  | 0.06-2 | 2 ^a^ | 266.7 ^a^ | 0,1,2,3,4,5 | 1 | - | 1 |  |  |
| ^47^2010/Nagel | *S. aureus* | CIP |  | ND |  | ND | - |  | - | - | 3.4*^b^ | - | No | 3 |
| ^48^2012/Nair | *P. aeruginosa* | NOR |  | 0.57 |  | 0.7 | - |  | - | - | 12^a^ | - | No | 0 |
| ^49^2008/Noguera | *K. pneumoniae* | CIP | multi | ND | no | 0.023-0.38 | 18.7* | 84.2* | - | - | - | - | No | 1 |
|  | *K. pneumoniae* | MOX |  |  |  | 0.047-0.5 | 2.3* | 9.6* | - | - | - | - |  |  |
|  | *K. pneumoniae* | LEV |  |  |  | 0.047-1 | 7.7* | 26.7* | - | - | - | - |  |  |
| ^50^1987/Ravizzola | *P. aeruginosa* | CIP | multi | ND | no | 0.5-4 | 3 ^a^ | 12.5 ^a^ | - | - | - | 0 | No | 6 |
| ^51^2007/Rodriguez | *M. chelonae* | MOX | multi | ND | no | 0.06-0.125 | >512 | >4266.7 | - | - | - | - | No | 3 |
|  | *M. fortuitum* | MOX |  |  |  | 0.06-0.125 | 128 | 2133.3 | - | - | - | - |  |  |
| ^52^2016/Song | *﻿E. coli* | CIP |  | ND |  | ND | - |  |  |  | 6.495*^a^ | - | No | 1 |
| ^53^2017/Sulyok | *M. bovis* | danOFL | multi | 0.5 | yes | 0.156 |  | 64.1 ^a^ | ND  ND | - | - | 1,2 | No | 6 |
|  | *M. bovis* | ENRO |  | 0.5 |  | 0.156 |  | 64.1 ^a^ |  | - | - | 1,2 |  |  |
|  | *M. bovis* | marbOFL |  | 0.5 |  | 0.312-0.625 | 16 ^a^ | 32.1 ^a^ |  | - | - | 1,2 |  |  |
| ^54^2018/Sun | *E. faecalis* | CIP | multi | ND, GP |  | 0.5-1 | 32 ^a^ | 128 ^a^ | 0,2 | - | - | 1 (tobr1mycin + Qs) | No | 1 |
|  | *E. faecalis* | LEV |  | ND, GP |  | 0.5-1 | 16 ^a^ | 256 ^a^ | 0,1,2 | - | - | 1 |  |  |
|  | *E. faecalis* | GAT |  | ND, GP |  | 0.25-0.5 | 16 ^a^ | 128 ^a^ | 0,1,2 | - | - | 1 |  |  |
| ^55^2008/Tanimoto | *P. aeruginosa* | CIP |  | 0.15 |  | 0.2 | - |  |  | - | 3.1**^a^ | 2 (meropenem) | No | 2 |
|  | *P. aeruginosa* | OFL |  | 0.3125 (clinical), 0.3125X for pare-al muta-s |  | 0.8 | - |  |  | - | 1.98* | 2 |  |  |
|  | *P. aeruginosa* | OFL |  | 0.156 |  | 1.6 | - |  |  | - | 3.6** | 2 |  |  |
| ^56^2011/Thi | *E. coli* | CIP |  | 0.5 | no | 0.12 | - |  |  | - | 2.1*^a^ | - | No | 1 |
| ^57^2015/Torres-  Barcelo | *P. aeruginosa* | CIP |  | 0.5 (IC50 dose) |  | 0.2 |  |  |  |  | 2.71*^b^ | - | No | 0 |
|  |  |  | multi | 0.5 | no | 0.2 |  | 8* |  |  |  |  |  |  |
| ^58^2001/ Venezia | *S. aureus* | CIP |  | 0.5 |  | 0.38-0.76 |  | - | - | - | 4200.2^a^ | - | No | 1 |
|  |  | CIP |  | 0.5 |  | 0.38 |  | - | - | - | 19230.77^b^ | - |  |  |
|  |  | LEV |  | 0.5 |  | 0.19-0.25 |  | - | - | - | 194.8^a^ | - |  |  |
|  |  | GAT |  | 0.5 |  | 0.064-0.125 |  | - | - | - | 33.6 ^a^ | - |  |  |
|  |  | MOX |  | 0.5 |  | 0.032-0.064 |  | - | - | - | 165.8 ^a^ | - |  |  |
| ^59^2016/Wasserman | *P. aeruginosa* | CIP | multi | 0.25 |  | 0.2 | ND |  | - | - | 11.3*^b^ mutation rate compared to co-rol passage, Table 3 | 0 | No | 0 |
| ^60^2006/Wickman | *E. faecalis* | CIP | single | ND,GP GP |  | 4 | NR | 4 | 0 | - | - | 1 | No | 1 |
|  | *E. faecalis* | CIP | multi | ND, GP |  |  |  | 32 | 1 | - | - | 1 |  |  |
|  | *E. faecalis* | LEV | single | ND, GP |  | 0.5 |  | 4 | 0 | - | - | 1 |  |  |
|  | *E. faecalis* | LEV | multi | ND, GP |  |  | 32 | 64 | 1,2 | - | - | 1 |  |  |
|  | *E. faecalis* | GAT | single | ND, GP |  | 0.25 | 2 | 4 | 0 | - | - | 1 |  |  |
|  | *E. faecalis* | GAT | multi | ND, GP |  |  | 16 | 32 | 0,1 | - | - | 1 |  |  |
|  | *E. faecalis* | MOX | single | ND, GP |  | 0.06-0.12 | NR | 2 | 0 | - | - | 1 |  |  |
|  | *E. faecalis* | MOX | multi | ND, GP |  |  | 33.3 | 133.3 | 1,2 | - | - | 1 |  |  |
|  | *E. faecium* | CIP | single | ND, GP |  | 2-4 | NR | 32 | 0 | - | - | 1 |  |  |
|  | *E. faecium* | CIP | multi | ND, GP |  |  | 4 | 64 | 0 | - | - | 1 |  |  |
|  | *E. faecium* | LEV | single | ND, GP |  | 2-4 | 1 | 2 | 0 | - | - | 1 |  |  |
|  | *E. faecium* | LEV | multi | ND, GP |  |  | NR | 32 | 0,1 | - | - | 1 |  |  |
|  | *E. faecium* | GAT | single | ND, GP |  | 2-4 | NR | 2 | 0 | - | - | 1 |  |  |
|  | *E. faecium* | GAT | multi | ND, GP |  |  | 2 | 8 | 0 | - | - | 1 |  |  |
|  | *E. faecium* | MOX | single | ND, GP |  | 2-4 | NR | 2 | 0 | - | - | 1 |  |  |
|  | *E. faecium* | MOX | multi | ND, GP |  |  | 2 | 16 | 0 | - | - | 1 |  |  |
| ^61^1999/Wu | *P. aeruginosa* | CIP | multi | 0.5 | yes | 0.5-8 |  | 12** **(median) | - | - | - | 2 | No | 2 |
| ^62^2011/Yim | *﻿S. typhimurium* | CIP |  | 0.2 |  | 0.05 | - |  |  | - | 1.83*^a^ | - | No | 1 |
|  | *S. typhimurium* | MOX |  | 0.25 |  | 0.2 | - |  |  | - | 1.78* | - |  |  |
|  | *S. typhimurium* | GAT |  | 0.2 |  | 0.05 | - |  |  | - | 2.27* | - |  |  |
|  | *S. typhimurium* | LEV |  | 0.125 |  | 0.08 | - |  |  | - | 1.87* | - |  |  |

^0^CIP= ciprofloxacin, NOR- norfloxacin, LOM – lomefloxacin, GEMI- Gemifloxacin, MOX- moxifloxacin, GAT- gatifloxacin, SPAR- sparfloxacin, TROV- trovafloxacin, ENRO- enrofloxacin, CLIN – clinafloxacin, OFL- ofloxacin, ENX- enoxacin, SITA- sitafloxacin, GREP- grepafloxacin, PEF- pefloxacin, GAR- garenoxacin

^1^Single exposure (single) or multi-step (multi) passaging

^2^ Exposure concentration (X times MIC), ND=not defined by MIC, GP= gradient plate

^3^ Is treatment- adjusted to change in MIC from previous passage, ND= not defined/stated

^4^ Minimum fold change in MIC reported, NR=no change reported, ^a^ = value after passage on drug-free media

^5^ Maximum fold change in MIC reported, NR=no change reported, ^a^ = value after passage on drug-free media

^6^ Induced mutations in QRDR, - = not tested, ND= not defined, # = # of mutations

^7^ If efflux was tested, is there are role, 1=yes, 0=no, - = not tested, ND= not defined/stated

^8^ Fold increase in Mutagenesis (fold-increase of frequency or rate), - = not tested, ND=no quantitative data, ^a^ = mutant/isolation frequency, ^b^= mutation rate

^9^ If selection or development of multi-drug resistance/cross-resistance was tested and observed at least once (≥2-fold increase), - = not tested, 0=No, 1=FQ, 2=other classes,

^10^ Risk of Bias score using non-weighted assessment- tool

* average (taken from less than 5 values or as stated in text)

** median (taken from over 5 values or as stated in text)

***mode (as stated in text)

**
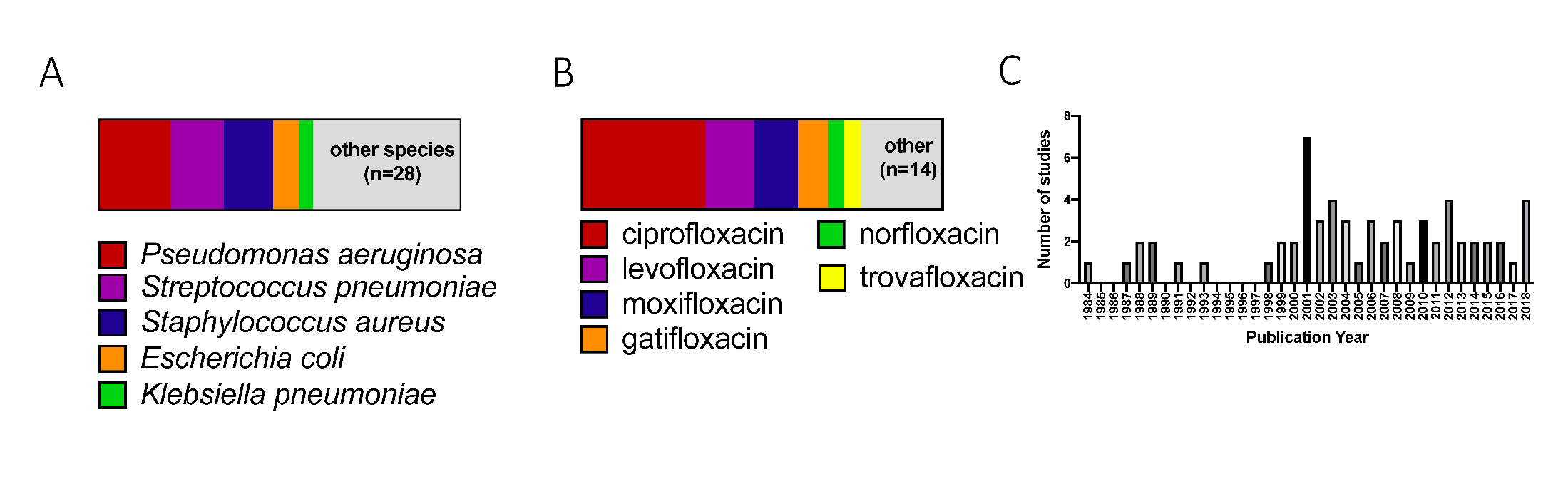
**

**Figure S1. Distribution of tested bacteria and fluoroquinolones in included studies show an enrichment for five types (displayed as % of total included studies)**

**(A)** Distribution of bacteria studied in included articles. Highlighted by color are the top 5 studied bacteria.  **(B)** Distribution of fluoroquinolones studied in included articles. Highlighted by color are the top 5 drugs studied. **(C)** Distribution of year of publication for studies included. 62 studies were included.


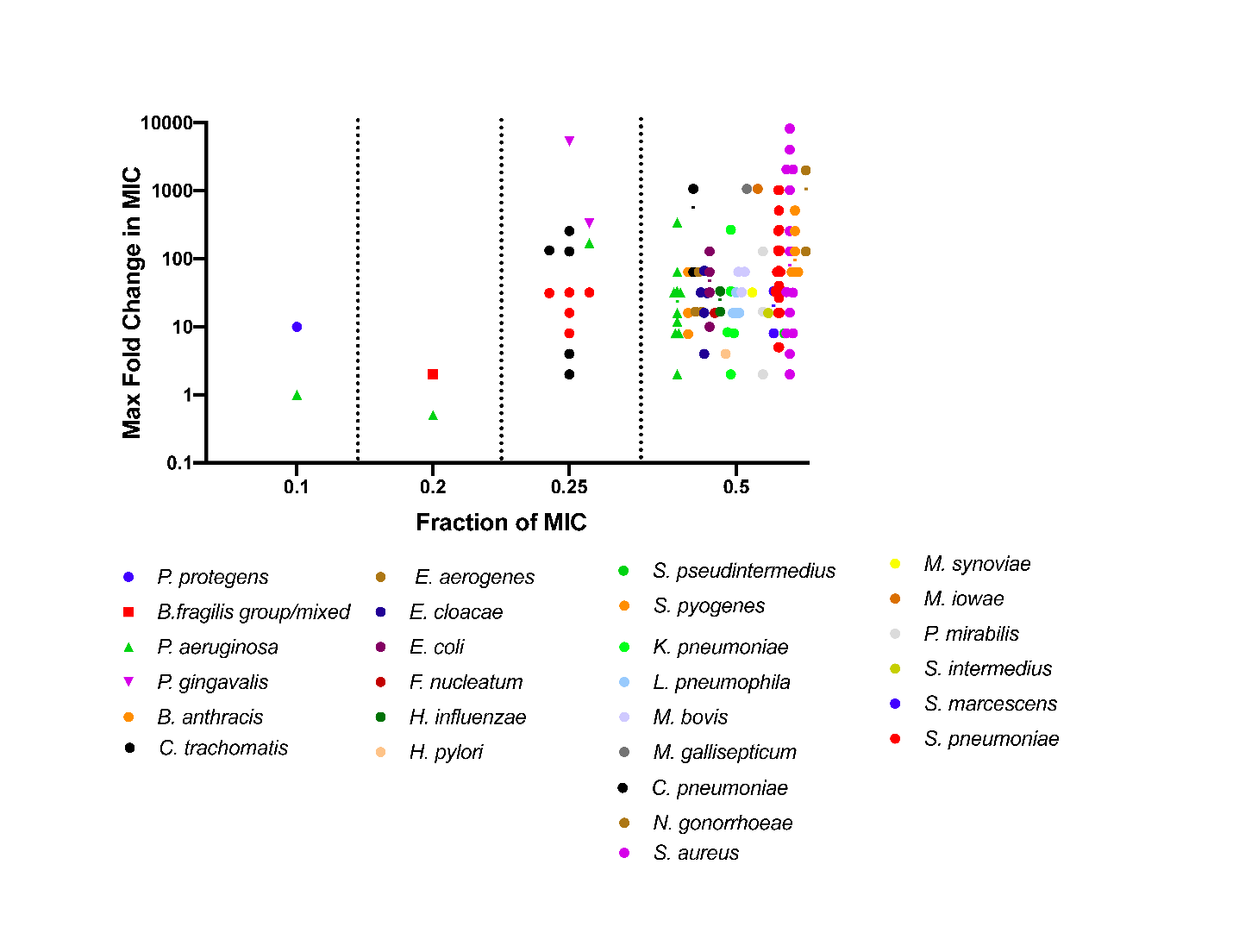


**Figure S2. Resistance by bacterial species**

Spread of maximum fold change in MIC, relative to parental strain, to exposure drug plotted against concentration of treatment. Each point represent one experimental test (bacteria-drug combination) and is colored by bacterial species

**
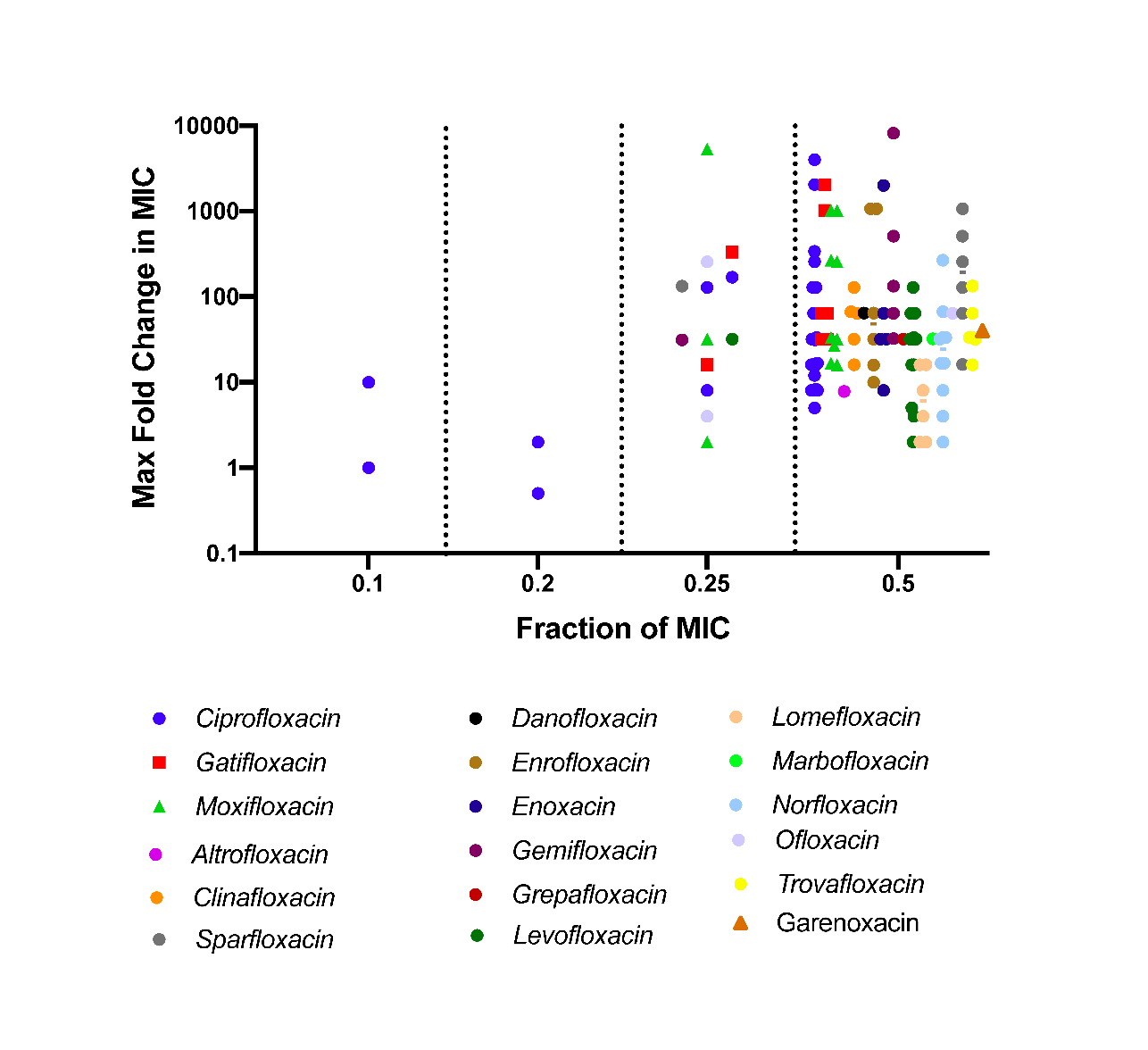
**

**Figure S3. Resistance by antibiotic**

Spread of maximum fold change in MIC, relative to parental strain, to exposure drug plotted against concentration of treatment. Each point represent one experimental test (bacteria-drug combination) and is colored by fluoroquinolone tested.


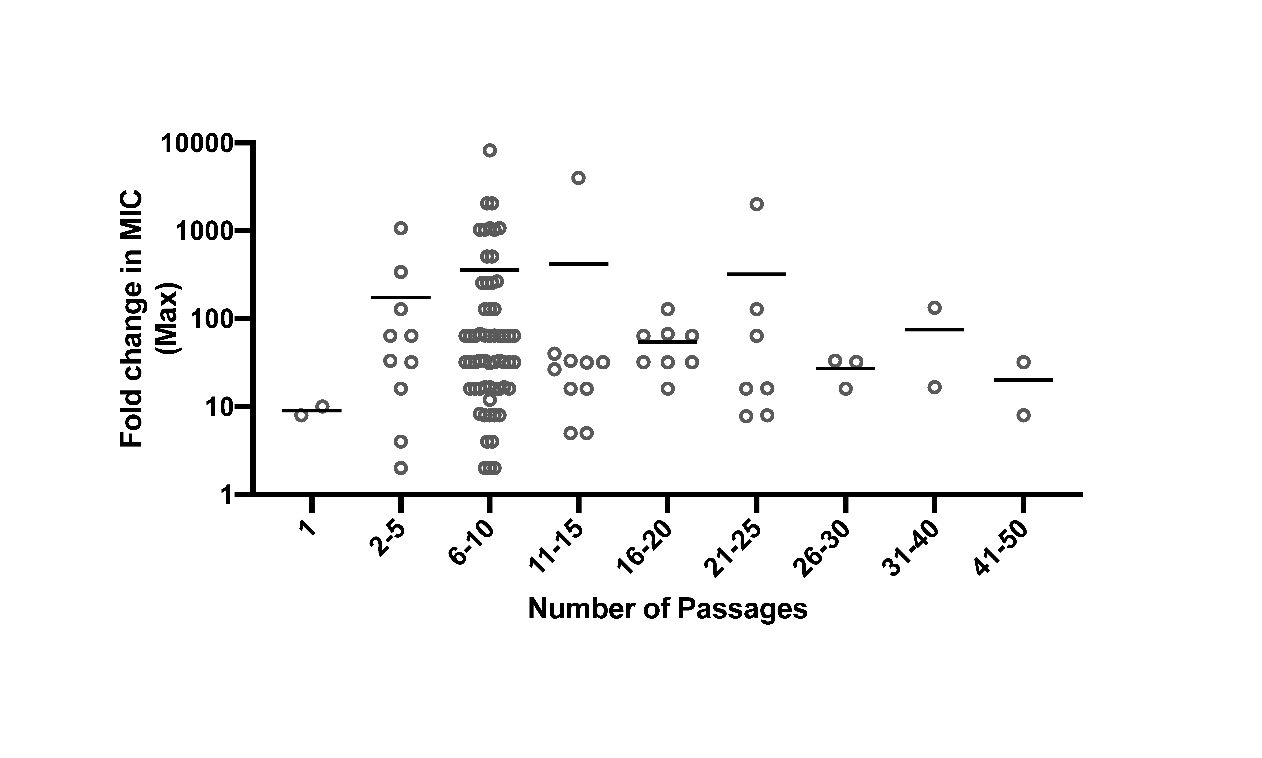


**Figure S4. Number of passages vs fold change in MIC**

Spread of maximum fold increase in MIC, relative to parental strain, plotted against number of passages for all studies with tests with fluoroquinolone exposure at 0.5X MIC. Each point represent one experimental test (bacteria-drug combination). Line represents mean.

References:

1. Ahmed MN, Porse A, Sommer MOA, *et al.* Evolution of Antibiotic Resistance in Biofilm and Planktonic Pseudomonas aeruginosa Populations Exposed to Subinhibitory Levels of Ciprofloxacin. *Antimicrob Agents Chemother* 2018; **62**: 1–12.

2. Aiassa V, Barnes AI, Smania AM, *et al.* Sublethal ciprofloxacin treatment leads to resistance via antioxidant systems in Proteus mirabilis. *FEMS Microbiol Lett* 2012; **327**: 25–32.

3. Aldridge KE, Henderberg A, Gebbia K, *et al.* Lomefloxacin, a new fluoroquinolone. Studies on in vitro antimicrobial spectrum, potency, and development of resistance. *Diagn Microbiol Infect Dis* 1989; **12**: 221–33.

4. Avrain L, Garvey M, Mesaros N, *et al.* Selection of quinolone resistance in Streptococcus pneumoniae exposed in vitro to subinhibitory drug concentrations. *J Antimicrob Chemother* 2007; **60**: 965–72.

5. Bai H, Du J, Hu M, *et al.* Analysis of mechanisms of resistance and tolerance of Escherichia coli to enrofloxacin. *Ann Microbiol* 2012; **62**: 293–8.

6. Barry AL, Jones RN. Cross-resistance among cinoxacin, ciprofloxacin, DJ-6783, enoxacin, nalidixic acid, norfloxacin, and oxolinic acid after in vitro selection of resistant populations. *Antimicrob Agents Chemother* 1984; **25**: 775–7.

7. Boos M, Mayer S, Fischer A, *et al.* In vitro development of resistance to six quinolones in Streptococcus pneumoniae, Streptococcus pyogenes, and Staphylococcus aureus. *Antimicrob Agents Chemother* 2001; **45**: 938–42.

8. I. B, T.B. E, H.I. PII, *et al.* In vitro resistance of Bacillus anthracis Sterne to doxycycline, macrolides and quinolones. *Int J Antimicrob Agents* 2001; **18**: 559–62.

9. Browne FA, Clark C, Bozdogan B, *et al.* Single and multi-step resistance selection study in Streptococcus pneumoniae comparing ceftriaxone with levofloxacin, gatifloxacin and moxifloxacin. *Int J Antimicrob Agents* 2002; **20**: 93–9.

10. Browne FA, Bozdogan B, Clark C, *et al.* Antipneumococcal activity of DK-507k, a new quinolone, compared with the activities of 10 other agents. *Antimicrob Agents Chemother* 2003; **47**: 3815–24.

11. Carman RJ, Woodburn MA. Effects of low levels of ciprofloxacin on a chemostat model of the human colonic microflora. *Regul Toxicol Pharmacol* 2001; **33**: 276–84.

12. Chow L, Waldron L, Gillings MR. Potential impacts of aquatic pollutants: sub-clinical antibiotic concentrations induce genome changes and promote antibiotic resistance. *Front Microbiol* 2015; **6**: 1–10.

13. Cottagnoud P, Entenza JM, Cottagnoud M, *et al.* Sub-inhibitory concentrations of vancomycin prevent quinolone-resistance in a penicillin-resistant isolate of Streptococcus pneumoniae. *BMC Microbiol* 2001; **1**: 1–4.

14. Dalhoff A, Schubert S. Dichotomous selection of high-level oxacillin resistance in Staphylococcus aureus by fluoroquinolones. *Int J Antimicrob Agents* 2010; **36**: 216–21.

15. Davies TA, Pankuch GA, Dewasse BE, *et al.* In vitro development of resistance to five quinolones and amoxicillin-clavulanate in Streptococcus pneumoniae. *Antimicrob Agents Chemother* 1999; **43**: 1177–82.

16. de Souza Filho JA, Diniz CG, Barbosa NB, *et al.* Morphological, biochemical, physiological and molecular aspects of the response of Fusobacterium nucleatum exposed to subinhibitory concentrations of antimicrobials. *Anaerobe* 2012; **18**: 566–75.

17. Dessus-babus S, Bebear CM, Charron A, *et al.* Sequencing of gyrase and topoisomerase IV quinolone-resistance-determining regions of Chlamydia trachomatis and characterization of quinolone-resistant mutants obtained In vitro. *Antimicrob Agents Chemother* 1998; **42**: 2474–81.

18. Efimochkina NR, Stetsenko V V., Bykova I V., *et al.* Studying the Phenotypic and Genotypic Expression of Antibiotic Resistance in Campylobacter jejuni under Stress Conditions. *Bull Exp Biol Med* 2018; **164**: 466–72.

19. Eick S, Schmitt A, Sachse S, *et al.* In vitro antibacterial activity of fluoroquinolones against Porphyromonas gingivalis strains. *J Antimicrob Chemother* 2004; **54**: 553–6.

20. Ferguson MI, Scott EM, Collier PS. Development of resistance to ciprofloxacin in nutrient-rich and nutrient- limited growth conditions in vitro by Pseudomonas aeruginosa isolates from patients with cystic fibrosis. *Antimicrob Agents Chemother* 1991; **35**: 2649–51.

21. Flatz L, Cottagnoud M, Kuhn F, *et al.* Ceftriaxone acts synergistically with levofloxacin in experimental meningitis and reduces levofloxacin-induced resistance in penicillin-resistant pneumococci. *J Antimicrob Chemother* 2004; **53**: 305–10.

22. French GLL, Ling J, Ling T, *et al.* Susceptibility of Hong Kong isolates of methicillin-resistant Staphylococcus aureus to antimicrobial agents. *J Antimicrob Chemother* 1988; **21**: 581–8.

23. Frenoy A, Bonhoeffer S. Death and population dynamics affect mutation rate estimates and evolvability under stress in bacteria de Visser A, ed. *PLoS Biol* 2018; **16**: e2005056.

24. Fung-Tomc J, Kolek B, Bonner DP, *et al.* Ciprofloxacin-induced, low-level resistance to structurally unrelated antibiotics in Pseudomonas aeruginosa and methicillin-resistant Staphylococcus aureus. *Antimicrob Agents Chemother* 1993; **37**: 1289–96.

25. Ganière JP, Médaille C, Limet A, *et al.* Antimicrobial activity of enrofloxacin against Staphylococcus intermedius strains isolated from canine pyodermas. *Vet Dermatol* 2001; **12**: 171–5.

26. Gautier-Bouchardon A V, Reinhardt AK, Kobisch M, *et al.* In vitro development of resistance to enrofloxacin, erythromycin, tylosin, tiamulin and oxytetracycline in Mycoplasma gallisepticum, Mycoplasma iowae and Mycoplasma synoviae. *Vet Microbiol* 2002; **88**: 47–58.

27. Gillespie SH, Basu S, Dickens AL, *et al.* Effect of subinhibitory concentrations of ciprofloxacin on Mycobacterium fortuitum mutation rates. *J Antimicrob Chemother* 2005; **56**: 344–8.

28. Gregoire N, Raherison S, Grignon C, *et al.* Semimechanistic pharmacokinetic-pharmacodynamic model with adaptation development for time-kill experiments of ciprofloxacin against Pseudomonas aeruginosa. *Antimicrob Agents Chemother* 2010; **54**: 2379–84.

29. Henderson-Begg SK, Livermore DM, Hall LMC. Effect of subinhibitory concentrations of antibiotics on mutation frequency in Streptococcus pneumoniae. *J Antimicrob Chemother* 2006; **57**: 849–54.

30. Iwao E, Yokoyama Y, Yamamoto K, *et al.* In vitro and in vivo anti- Helicobacter pylori activity of Y-904, a new fluoroquinolone. *J Infect Chemother* 2003; **9**: 165–71.

31. Jonas D, Engels I, Hartung D, *et al.* Development and mechanism of fluoroquinolone resistance in Legionella pneumophila. *J Antimicrob Chemother* 2003; **51**: 275–80.

32. Jones ME, Boenink NM, Verhoef J, *et al.* Multiple mutations conferring ciprofloxacin resistance in Staphylococcus aureus demonstrate long-term stability in an antibiotic-free environment. *J Antimicrob Chemother* 2000; **45**: 353–6.

33. Jørgensen KM, Wassermann T, Jensen PØO, *et al.* Sublethal ciprofloxacin treatment leads to rapid development of high-level ciprofloxacin resistance during long-term experimental evolution of Pseudomonas aeruginosa. *Antimicrob Agents Chemother* 2013; **57**: 4215–21.

34. Kaskatepe B, Yildiz S. In Vitro Development of Resistance to Subinhibitory Concentrations of Ciprofloxacin and Levofloxacin in Uropathogen Escherichia coli. *J PURE Appl Microbiol* 2013; **7**: 1431–5.

35. Kim MJ, Yun HJ, Kang JW, *et al.* In vitro development of resistance to a novel fluoroquinolone, DW286, in methicillin-resistant Staphylococcus aureus clinical isolates. *J Antimicrob Chemother* 2003; **51**: 1011–6.

36. Koeth LM, Good CE, Saunders KA, *et al.* Streptococcus pneumoniae in vitro development of resistance to amoxicillin/clavulanic acid, cefaclor, levofloxacin and azithromycin. *Int J Antimicrob Agents* 2004; **24**: 144–9.

37. Korting HC, Lukacs A. Decrease of antimicrobial susceptibility of gonococci to newer quinolones after repeated exposition to subinhibitory concentrations in vitro. *Infection* 1989; **17**: S6–10.

38. Kosowska-Shick K, Credito K, Pankuch GA, *et al.* Antipneumococcal activity of DW-224a, a new quinolone, compared to those of eight other agents. *Antimicrob Agents Chemother* 2006; **50**: 2064–71.

39. Kosowska-Shick K, Clark C, Credito K, *et al.* In vitro capability of faropenem to select for resistant mutants of Streptococcus pneumoniae and Haemophilus influenzae. *Antimicrob Agents Chemother* 2008; **52**: 748–52.

40. Kumari H, Balasubramanian D, Zincke D, *et al.* Role of pseudomonas aeruginosa AmpR on β-lactam and non-β-lactam transient cross-resistance upon pre-exposure to subinhibitory concentrations of antibiotics. *J Med Microbiol* 2014; **63**: 544–55.

41. Mesak LR, Davies J. Phenotypic changes in ciprofloxacin-resistant Staphylococcus aureus. *Res Microbiol* 2009; **160**: 785–91.

42. Modak SM, Sampath L, Fox CLJ. Combined topical use of silver sulfadiazine and antibiotics as a possible solution to bacterial resistance in burn wounds. *J Burn Care Rehabil* 1988; **9**: 359–63.

43. Mohamed MF, Hammac GK, Guptill L, *et al.* Antibacterial Activity of Novel Cationic Peptides against Clinical Isolates of Multi-Drug Resistant Staphylococcus pseudintermedius from Infected Dogs. *PLoS One* 2014; **9**: 1–20.

44. Morrissey I, Salman H, Bakker S, *et al.* Serial passage of Chlamydia spp. in sub-inhibitory fluoroquinolone concentrations. *J Antimicrob Chemother* 2002; **49**: 757–61.

45. Nagai K, Davies TA, Pankuch GA, *et al.* In vitro selection of resistance to clinafloxacin, ciprofloxacin, and trovafloxacin in Streptococcus pneumoniae. *Antimicrob Agents Chemother* 2000; **44**: 2740–6.

46. Nagai K, Davies TA, Dewasse BE, *et al.* Single- and multi-step resistance selection study of gemifloxacin compared with trovafloxacin, ciprofloxacin, gatifloxacin and moxifloxacin in Streptococcus pneumoniae. *J Antimicrob Chemother* 2001; **48**: 365–74.

47. Nagel M, Reuter T, Jansen A, *et al.* Influence of ciprofloxacin and vancomycin on mutation rate and transposition of IS256 in Staphylococcus aureus. *Int J Med Microbiol* 2011; **301**: 229–36.

48. Nair CG, Chao C, Ryall B, *et al.* Sub-lethal concentrations of antibiotics increase mutation frequency in the cystic fibrosis pathogen Pseudomonas aeruginosa. *Lett Appl Microbiol* 2013; **56**: 149–54.

49. Noguera O, Rodriguez JC, Cremades R, *et al.* [In vitro generation of mutants of Klebsiella pneumoniae following exposure to fluorquinolones. Relationship with the presence of extended spectrum betalactamases]. *Rev Esp Quimioter* 2008; **21**: 180–3.

50. Ravizzola G, Pirali F, Paolucci A, *et al.* Reduced virulence in ciprofloxacin-resistant variants of Pseudomonas aeruginosa strains. *J Antimicrob Chemother* 1987; **20**: 825–9.

51. Rodriguez JC, Flores E, Escribano I, *et al.* Generation of resistant mutants of Mycobacterium chelonae and Mycobacterium fortuitum after exposure to subinhibitory concentrations of clarithromycin and moxifloxacin. *J Chemother* 2007; **19**: 599–601.

52. Song LY, Goff M, Davidian C, *et al.* Mutational consequences of ciprofloxacin in Escherichia coli. *Antimicrob Agents Chemother* 2016; **60**: 6165–72.

53. Sulyok KM, Kreizinger Z, Wehmann EE, *et al.* Mutations Associated with Decreased Susceptibility to Seven Antimicrobial Families in Field and Laboratory-Derived Mycoplasma bovis Strains. *Antimicrob Agents Chemother* 2017; **61**: e01983-16.

54. Sun Y, Lu H, Zhang X, *et al.* Phenotype and genotype alteration during adaptive evolution of Enterococcus faecalis to antimicrobials. *Infect Genet Evol* 2018; **62**: 80–5.

55. Tanimoto K, Tomita H, Fujimoto S, *et al.* Fluoroquinolone enhances the mutation frequency for meropenem-selected carbapenem resistance in Pseudomonas aeruginosa, but use of the high-potency drug doripenem inhibits mutant formation. *Antimicrob Agents Chemother* 2008; **52**: 3795–800.

56. Do Thi T, López E, Rodríguez-Rojas A, *et al.* Effect of recA inactivation on mutagenesis of Escherichia coli exposed to sublethal concentrations of antimicrobials. *J Antimicrob Chemother* 2011; **66**: 531–8.

57. Torres-Barcelo C, Kojadinovic M, Moxon R, *et al.* The SOS response increases bacterial fitness, but not evolvability, under a sublethal dose of antibiotic. *Proceedings Biol Sci* 2015; **282**: 20150885.

58. Venezia RA, Domaracki BE, Evans AM, *et al.* Selection of high-level oxacillin resistance in heteroresistant Staphylococcus aureus by fluoroquinolone exposure. *J Antimicrob Chemother* 2001: 375–81.

59. Wassermann T, Jørgensen KM, Ivanyshyn K, *et al.* The phenotypic evolution of Pseudomonas aeruginosa populations changes in the presence of subinhibitory concentrations of ciprofloxacin. *Microbiology* 2016; **162**: 865–75.

60. Wickman PA, Black JA, Smith Moland ES, *et al.* In vitro development of resistance to DX-619 and other quinolones in enterococci. *J Antimicrob Chemother* 2006; **58**: 1268–73.

61. Wu Y-LL, Scott EMM, Po AL, *et al.* Development of resistance and cross-resistance in Pseudomonas aeruginosa exposed to subinhibitory antibiotic concentrations. *APMIS* 1999; **107**: 585–92.

62. Yim G, McClure J, Surette MG, *et al.* Modulation of Salmonella gene expression by subinhibitory concentrations of quinolones. *J Antibiot (Tokyo)* 2011; **64**: 73–8.
